# Hydrogen-Bonding-Aided Fabrication of Wood Derived Cellulose Scaffold/Aramid Nanofiber into High-Performance Bulk Material

**DOI:** 10.3390/ma14185444

**Published:** 2021-09-20

**Authors:** Xiaoshuai Han, Weijie Wu, Jingwen Wang, Zhiwei Tian, Shaohua Jiang

**Affiliations:** Jiangsu Co-Innovation Center of Efficient Processing and Utilization of Forest Resources, International Innovation Center for Forest Chemicals and Materials, College of Materials Science and Engineering, Nanjing Forestry University, Nanjing 210037, China; hxs141424@njfu.edu.cn (X.H.); 18751958661@163.com (W.W.); w571948261jw@163.com (J.W.); tianviatian@163.com (Z.T.)

**Keywords:** wood, cellulose scaffolds, aramid nanofibers, densification, hydrogen bonding, mechanical properties

## Abstract

Preparing a lightweight yet high-strength bio-based structural material with sustainability and recyclability is highly desirable in advanced applications for architecture, new energy vehicles and spacecraft. In this study, we combined cellulose scaffold and aramid nanofiber (ANF) into a high-performance bulk material. Densification of cellulose microfibers containing ANF and hydrogen bonding between cellulose microfibers and ANF played a crucial role in enhanced physical and mechanical properties of the hybrid material. The prepared material showed excellent tensile strength (341.7 MPa vs. 57.0 MPa for natural wood), toughness (4.4 MJ/m^3^ vs. 0.4 MJ/m^3^ for natural wood) and Young’s modulus (24.7 GPa vs. 7.2 GPa for natural wood). Furthermore, due to low density, this material exhibited a superior specific strength of 285 MPa·cm^3^·g^−1^, which is remarkably higher than some traditional building materials, such as concrete, alloys. In addition, the cellulose scaffold was infiltrated with ANFs, which also improved the thermal stability of the hybrid material. The facile and top-down process is effective and scalable, and also allows one to fully utilize cellulose scaffolds to fabricate all kinds of advanced bio-based materials.

## 1. Introduction

Structural materials with lightweight yet superior mechanical performance have always been a research hotspot in the field of engineering, construction and architecture. Natural wood is a very important kind of structural materials for building and furniture due to its low cost, easy processing and abundant [1,2,3]. However, the physical and mechanical performance of natural wood cannot meet the demand for advanced engineering materials [4,5,6]. Based on this, quality optimization process for natural wood is an effective strategy to achieve the high value application of wood [7,8,9].

In recent years, partial or full delignification pretreatment while retaining hierarchical structure of the natural wood has been a strong focus due to their excellent mechanical properties and promising serve as functionality for advanced composite materials [10,11,12,13,14,15]. After delignification, the following processes, such as polymer matrix impregnation and densification processes, were used to fabricate structure-retaining cellulosic composites with improved mechanical performance and novel functionalities [16,17,18,19,20,21,22,23] such as Yano et al. impregnated phenol formaldehyde (PF) resin into delignified wood, following by compression to different density levels and studied mechanical properties. The results showed the bending strength up to 670 MPa and elastic modulus up to 62 GPa [24]. Frey et al. investigated the mechanical performance of delignified spruce wood under tensile loading. The largest densification resulted in a highest density of around 1.1 g/cm^3^, inducing ~270 MPa tensile strength and ~35 GPa elastic modulus even without inserting any matrix for resisting force [11]. For densification approaches, our previous research has proved that samples compressed at higher moisture content (MC) (18% MC) had a better mechanical performance. More importantly, the strength and fracture toughness, which is generally mutually exclusive performance can be simultaneously improved due to water molecule-induced hydrogen bonding between aligned cellulose nanofibers [25,26,27]. Based on above conclusions, the wood derived cellulose scaffold retaining inherent hierarchical structure can be processed into superior mechanical advanced material by inserting substance that can trigger more hydrogen bonding between cellulose microfibers under compression at certain moisture content.

As a kind of novel nanofibers, aramid nanofiber (ANF) has always been a research focus. It is usually obtained from commercial poly(p-phenylene terephthalamide) (PPTA) threads by “top-down” approach [28,29]. Similarly with other nanofiber, ANF exhibits anisotropic properties, nanoscale morphologies, large aspect ratio and high specific surface area. More impressively, ANF also inherits excellent mechanical performance, water resistance and thermal stability [30,31,32,33]. So far, ANF has been extensively applied in the reinforcement for polymer composites, such as polyvinyl alcohol (PVA) [34], poly(ethyleneoxide) (PEO) [35], epoxy resin [36] and cellulose nanofiber composite [37].

Inspired by the superior performances of cellulosic scaffold and ANF, herein, we reported a versatile strategy to fabricate high-performance material with superior mechanical performance and good thermal stability via vacuum dipping technique and densification process.

## 2. Materials and Methods

### 2.1. Materials and Chemicals

Basswood with dimensions of 50 × 30 × 10 mm^3^ (longitudinal × tangential × radial) was used in this work. Kevlar 29 yarn, produced by DuPont, was provide from Changzhou Hualike New Materials Co., Ltd. (Jiangsu, China) Sodium chlorite (NaClO_2_, 80%), sulfuric acid (H_2_SO_4_, 72%), acetic acid (HAc, 99.7%), sodium hydroxide (NaOH, 97%), dimethyl sulfoxide (DMSO), potassium hydroxide (KOH), acetone (99.8%) and hexane (99.8%) were purchased from Fisher Scientific (Waltham, MA, USA). All chemicals were used as received without further purification. Deionized (DI) water was used for whole experimental process.

### 2.2. Preparation of Aramid Nanofiber (ANF) Suspension

A 2 mg/mL ANF suspension was prepared referring to previous reports [38,39]. Specifically, 1 g of bulk Kevlar 29 and 0.5 g KOH were added into 500 mL DMSO, which was then magnetically stirred for 1 week under ambient conditions forming a dark red, viscous suspension of ANF (Figure S1).

### 2.3. Fabrication of Aligned Cellulose Microfiber/ANF Hybrid Material

In order to obtain aligned cellulose scaffold (CS), natural basswood (NW) sample was chemically treated to remove lignin and hemicellulose according to our previous work (Figure S2) [25,40]. The obtained CS was infiltrated with 0.5 mg/mL ANF suspension. Then, the impregnated CS was subjected to solvent-exchange method to remove DMSO, following by air drying (CS_AD_) to 18% MC. Finally, above sample was densified along radial direction using a hot press (ZG-50TSD, Dongguan Zhenggong Electromechanical Equipment Technology Co., Ltd., Dongguan, China) under 35 MPa pressure for 10 min to fabricate aligned cellulose microfiber/ANF hybrid material (CCS_AD-ANF_). After densification, the densified sample was dried at 80 °C for 24 h and then cooled to room temperature for further characterization.

### 2.4. Chemical Component Analysis

The cellulose, hemicellulose and lignin contents of NW and CS was tested based on standard TAPPI T 222 om-2 method [41]. Specifically, 200 mg of dry powdered sample was fully acid-hydrolyzed using 3 mL 72% H_2_SO_4_, following by adding 112 mL DI water and was then autoclaved at 121 °C for 1 h. The acid-insoluble lignin was filtered, oven-dried and weighed. The acid-soluble lignin was determined based on the absorbance at 205 nm using UV spectrophotometer. The sugars were analyzed by Agilent 1200 high-performance liquid chromatograph (HPLC) using a Bio-Rad Aminex 87H column. All assays were carried out in triplicate.

### 2.5. Density and Porosity Tests

The densities of NW, CS_AD_ and CCS_AD-ANF_ were calculated from the ratios of mass to volume. The porosities of these three samples were determined as follows:

(1) $$\;\mathrm{Porosity}\;\left(\%\right)=\left(1-\frac{\rho_{a}}{\rho_{c}}\right)\times 100\%$$

where $\rho_{a}$ is the density of the NW, CS_AD_ and CCS_AD-ANF_ samples and $\rho_{c}$ is the density of pure cellulosic specimen, taken as 1.5 g cm^−3^ [42].

### 2.6. Fourier Transform Infrared (FTIR) Analysis

FTIR spectrum of the samples was obtained using an Attenuated Total Reflectance Fourier Transform Infrared spectrometer (VERTEX 80V, Bruker, Germany) from 4000 to 400 cm^−1^ at a spectral resolution of 4 cm^−1^ with a total of 128 scans.

### 2.7. Scanning Electron Microscopy (SEM) Analysis

The morphologies of NW, CS_AD_ and CCS_AD-ANF_ were characterized using Phenom XL-SE-G2 Desktop Scanning Electron Microscope (Phenom-World BV, Eindhoven, Netherlands), SED mode and accelerating voltage of 10 kV. The samples were sliced using cutall microtome and then oven-dried, following by sputter-coated with Au target prior to observation.

### 2.8. Mechanical Performance Tests

The mechanical properties of all samples were tested using the 3365 universal testing machine (Instron Test Equipment Trading Co., Ltd., Hangzhou, China). Based on a standard measurement [43], all samples were produced in dog-bone shape specimens with 50 mm length × 2 mm width (middle section) dimensions. The specimens were fixed at both ends using a mechanical clamp and stretched along the longitudinal direction until failure. The testing was conducted using a 5 kN load cell at 2 mm∙min^−1^ rate under room conditions. At least 15 specimens were tested for every sample and average value and standard deviation were calculated.

### 2.9. Thermogravimetric Analysis (TGA)

To investigate the thermal stability of NW, CS_AD_ and CCS_AD-ANF_, roughly 5 mg of powdered samples were analyzed using a thermal analyzer (STA 449F3, NETZSCH, Selb, Germany). The test was performed from room temperature to 800 °C at a heating rate of 10° min^−1^ under nitrogen atmosphere (a flow of 20 mL/min).

### 2.10. X-ray Diffraction (XRD) Analysis

The XRD analysis was carried out through a Rigaku Ultima IV (Tokyo, Japan) (CuKα radiation with graphite monochromator, 40 kV and 30 mA). The patterns were obtained between 5° and 90° 2θ with 0.02° steps and scan speed of 10° min^−1^. The degree of crystallinity (Cr) was calculated using the empirical formular by Segal et al. [44].

(2) $$\;\mathrm{Cr}\;\left(\%\right)=\frac{I_{200}-I_{am}}{I_{200}}\times 100\%$$

where $I_{200}$ represented the peak at the maximum intensity that relates to the (200) lattice plane and $I_{am}$ is the minimum intensity value between the highest two peaks.

## 3. Results

**Figure 1:**
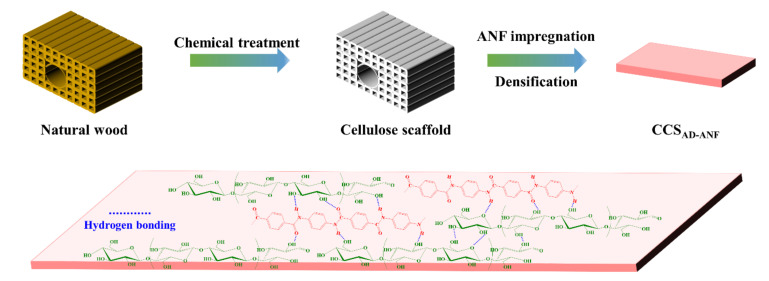
The preparation process of a high-performance cellulosic bulk material.

Figure 1 shows the complete process of preparing a high-performance cellulosic bulk material with the participation of ANF. Specifically, the material was fabricated following a three-step process including (1) delignification to remove most linin and some hemicelluloses, which can explore more cellulose microfibers, increasing hydrogen bonding sites; (2) CS was impregnated using ANF, which will take part in subsequent hydrogen bonding assembly of high-performance cellulosic materials; and (3) mechanical compression of infiltrated cellulosic materials with 18% moisture content to induce more hydrogen bonding for enhanced mechanical properties.

**Figure 2:**
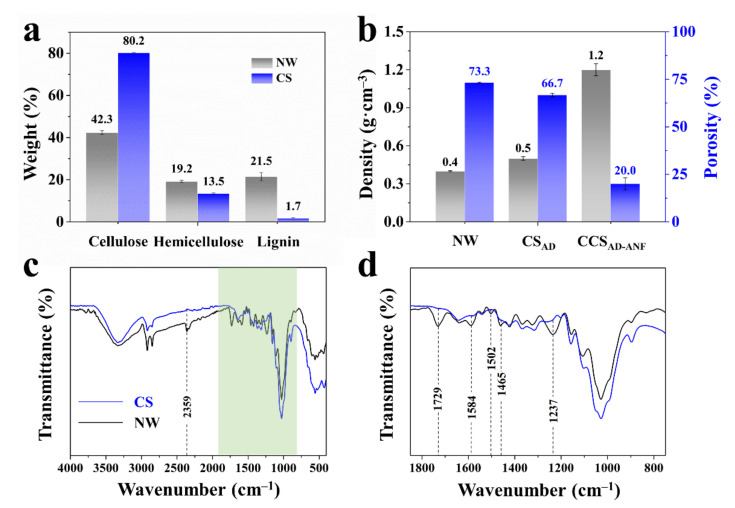
The comparison of cellulose, hemicellulose and lignin content in NW and CS (**a**). Density and porosity of NW, CS_AD_ and CCS_AD-ANF_ (**b**). FTIR spectra of NW (**c**) and CS (**d**).

In the microstructure of wood, the lignin plays a role of adhesive, which sticks cellulose and hemicellulose together. However, its existence decreased the accessibility of cellulose, restricting possible functionalization. So, the natural basswood was subjected to sodium chlorite (NaClO_2_) and sodium hydroxide (NaOH) treatment to remove lignin and hemicellulose. Results from the delignification process showed that the delignified wood contained 80.2% cellulose, 13.5% hemicellulose and 1.7% lignin, demonstrating 38.8% hemicellulose and 93.1% lignin removal (Figure 2a). In addition, samples of each stage under preparation showed different physical performance. Compared to NW, the CS_AD_ exhibited slightly increased density (0.5 vs. 0.4 g cm^−3^ for NW) and smaller porosity (66.7 vs. 73.3% for NW) due to air shrinkage (Figure 2b). The result of FTIR spectra of NW and CS also echoed the component analysis as shown in Figure 2c,d. For NW, the presence of 1584 cm^−1^ and 1502 cm^−1^ (C=C stretching vibration of the aromatic rings) represents the typical structure of lignin, while the band locating in 1237 cm^−1^ belongs to the C-O stretching of the aromatic rings. In addition, the peak at 1729 cm^−1^ is ascribed to unconjugated carbonyl C=O in hemicellulose. After NaClO_2_ and NaOH treatment, there are not these characteristic peaks for lignin and hemicellulose in CS sample.

**Figure 3:**
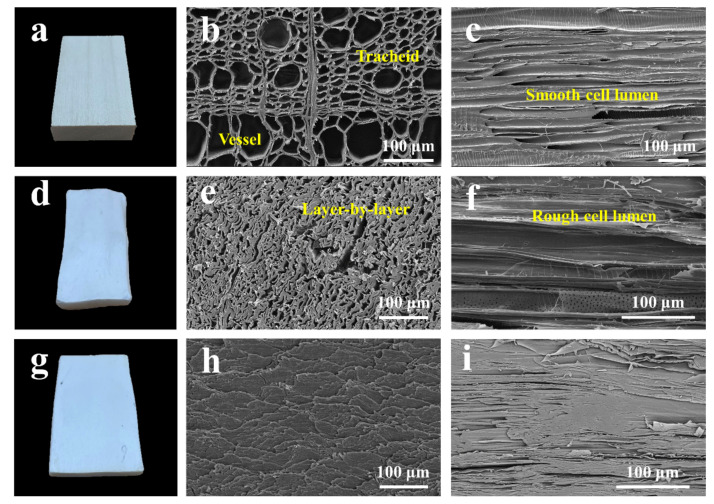
Photographs of (**a**) NW, (**d**) CS_AD_ and (**g**) CS_AD-ANF_. Transverse SEM images of (**b**) NW, (**e**) CS_AD_ and (**h**) CCS_AD-ANF_. Longitudinal SEM images of (**c**) NW, (**f**) CS_AD_ and (**i**) CCS_AD-ANF_.

The morphologies of NW, CS_AD_ and CCS_AD-ANF_ were shown in Figure 3. Due to drying shrinkage and densification, the thickness of CS_AD_ and CCS_AD-ANF_ gradually decreased in macroscopic level (Figure 3a,d,g). In case of SEM, the transverse section of NW looks like honeycomb, comprising of vessel and tracheid cells (Figure 3b and Figure S3a,b). The longitudinal microstructure of vessel and tracheid cells of NW is clear and smooth (Figure 3c and Figure S3c,d). After delignification, ANF impregnation and air-drying, the intrinsic honeycomb structure was destroyed and wood cell walls approached each other to form layer-by-layer structure (Figure 3e and Figure S4). In addition, the longitudinal section SEM image of CS_AD_ showed that the wood cells of vessel and tracheid became rough and appeared some aligned cellulose microfibers (Figure 3f and Figure S5). After absorbing certain water vapor (18% MC), CS_AD_ samples were subjected to compression under 35 MPa pressure. Compared to NW and CS_AD_, transverse section images of CCS_AD-ANF_ exhibited very dense and hard microstructure: wood cell lumen disappeared and cell walls closely bonded together (Figure 3h,i and Figure S6). The underlying cause for the dense and hard microstructure could be explained in three aspects. Firstly, a certain moisture content can effectively soften stiff cellulose microfibers within wood cell walls and water molecule can play a bridge role of hydrogen bonding between cellulose microfibers under compression. Secondly, densification process can obviously eliminate steric hindrance, inducing wood cell walls and cellulose microfibers to tightly intertwine and densely pack together by forming hydrogen bonding [25]. Thirdly, the impregnated ANF can fill up to void gap under compression and it also assist cellulose microfibers to form more hydrogen bonding between amide-group and hydroxyl groups. The above three factors played a significant role in forming dense and hard structure of CCS_AD-ANF_, which will help to improve the mechanical properties of product.

**Figure 4:**
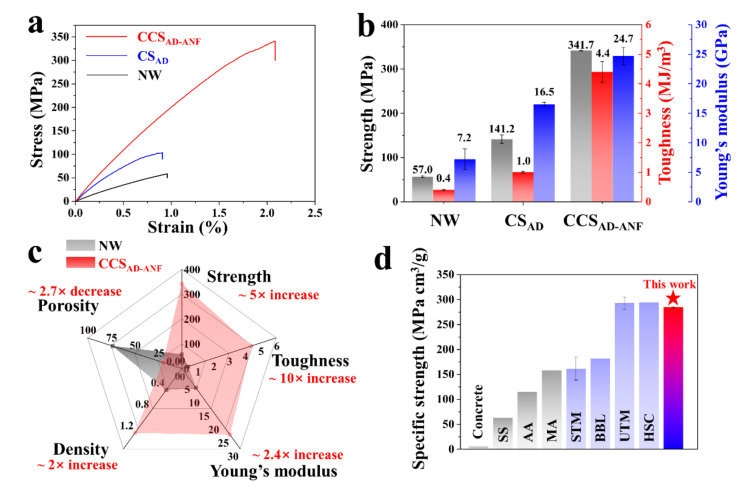
Mechanical performance of NW, CS_AD_ and CCS_AD-ANF_. (**a**) Tensile stress-strain curves for NW, CS_AD_ and CCS_AD-ANF_. (**b**) Tensile strength, toughness and Young’s modulus values derived from the tensile stress-strain curves of NW, CS_AD_ and CCS_AD-ANF_. (**c**) Comparison of the physical and mechanical properties of NW and CCS_AD-ANF_. (**d**) Comparison of the specific strength of CCS_AD-ANF_, concrete, stainless steel (304, SS), aluminum alloy (6061-T6, AA), magnesium alloy (MA), strong and tough material (STM), bio-based laminate (BBL), ultrastrong and tough material (UTM) and high-strength composite (HSC).

The mechanical properties of NW, CS_AD_ and CCS_AD-ANF_ are presented in Figure 4. Compared to NW, the CS_AD_ sample possessed improved strength (141.2 vs. 57.0 MPa), toughness (1.0 vs. 0.4 MJ/m^3^) and Young’s modulus (16.5 vs. 7.2 GPa) (Figure 4b). The enhancement of mechanical performance was attributed to water-induced hydrogen bonding between cellulose microfibers within CS cell walls. As for the CCS_AD-ANF_, the tensile strength (341.7 MPa), toughness (4.4 MJ/m^3^), Young’s modulus (24.7 GPa) and density (1.2 g/cm^3^) were more than 5, 10, 2.4, 2 times higher than those of NW, respectively (Figure 4b,c), which is consistent with the SEM images result. Both the microscopic structure and macroscopic mechanical properties of the NW, CS_AD_ and CCS_AD-ANF_ implied that lignin removal facilitated hydrogen bonding between cellulose microfibers, resulting in mechanical performance improvement. More importantly, the physical and mechanical properties were strikingly enhanced due to ANF impregnation. The ANF can take part in hydrogen bonding assembly between cellulose microfibers and it also can be inserted into micro defect under compression, resulting in very dense structure and excellent physical and mechanical performance. The specific strength is a kind of very important index in practical application. A comparison of specific tensile strength with conventional construction materials and lignocellulosic structural materials, including concrete, stainless steel (304, SS), aluminum alloy (6061-T6, AA), magnesium alloy (MA), strong and tough material (STM) [40], bio-based laminate (BBL) [19], ultrastrong and tough material (UTM) [25] and high-strength composite (HSC) [16] was plotted in Figure 4d. The specific strength of CCS_AD-ANF_ is significantly greater than that of traditional building materials and it even can match high-performance wooden structural composites. In addition, the specific strength is a very necessary benefit for the applications in areas, such as new energy vehicles, spacecraft, where lightweight high strong properties are required.

**Figure 5:**
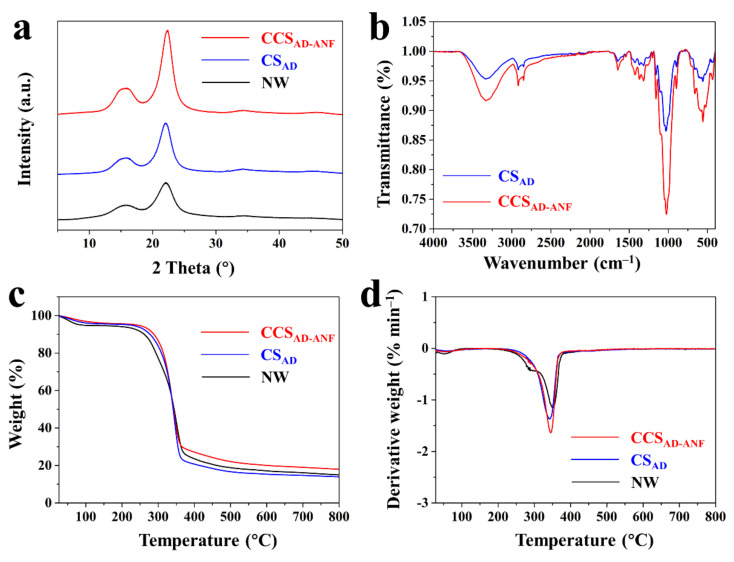
(**a**) XRD patterns of NW, CS_AD_ and CCS_AD-ANF_. (**b**) FTIR curves of CS_AD_ and CCS_AD-ANF_. TGA (**c**) and DTG (**d**) curves of NW, CS_AD_ and CCS_AD-ANF_.

The XRD patterns of NW, CS_AD_ and CCS_AD-ANF_ were shown in Figure 5a. Comparing the three curves, the peak position did not change, indicating that the delignification and ANF modification did not destroy the crystalline structure of cellulose. However, the significant difference of these three samples was the crystalline degree values: the Cr of NW, CS_AD_ and CCS_AD-ANF_ were 37.8%, 56.5% and 67.4%, respectively. The delignification process removed amorphous lignin and hemicellulose and preserved the crystal cellulose. Moreover, the high crystalline ANF impregnation endowed the CCS_AD-ANF_ with high crystallinity. High crystallinity can help improve mechanical properties of samples and the excellent mechanical performance of CCS_AD-ANF_ is consistent with the information reflected by the XRD results. The strong interactions between cellulose microfibers and ANF were reflected by FTIR in Figure 5b. The absorption peaks of about 3300 cm^−1^ and 1640 cm^−1^ are corresponded to the N-H and C=O stretching vibration of ANF, respectively [45,46]. Figure 5b showed that the N-H and C=O absorption peaks of the CCS_AD-ANF_ was significantly increased compared with CS_AD_ sample, indicating the ANF was successfully infiltrated into CS structure. In addition, the N-H and C=O chemical signal that differentiates the CS_AD_ from CCS_AD-ANF_ is the apparently upshift and the higher energy of these molecular vibrations is related to the stronger hydrogen bonds in the material [32,34]. Meanwhile, there’s a blue-shift of CCS_AD-ANF_ sample in 3300 cm^−1^ absorption peak, which can be ascribed to new hydrogen bonding formed between N-H groups of ANF and the O-H groups of cellulose microfibers (Figure S7).

The TG and DTG curves of NW, CS_AD_ and CCS_AD-ANF_ were presented in Figure 5c,d. The natural wood is commonly vulnerable to thermal decomposition and its three main components (cellulose, hemicellulose and lignin) have different thermal stability due to their inherent structure [47,48]. Typically, the pyrolysis of hemicellulose occurred at 220–315 °C and the thermal degradation of cellulose mainly happened at 315–400 °C. Meanwhile, the lignin has a pyrolysis between 150 °C and 600 °C. Due to hemicellulose and lignin removal, the CS_AD_ samples had a better thermal stability than NW between 220–315 °C. It was noted that the CCS_AD-ANF_, exhibited a better thermal stability. This was mainly due to ANF unique inherent microstructure, which has linear benzene ring structure, intermolecular hydrogen bonds and good crystallinity [49,50]. In addition, the initial weight loss before 220 °C was also attributed to the evaporation of free water and adsorbed water. The compression process of CS_ANF_ under 18% moisture content can make the free water molecule become structural water by taking part in hydrogen bonding between hydroxyl groups (CS) and amide groups (ANF). So, the CCS_AD-ANF_ samples had a better thermal stability than NW and CS_AD_ samples before 220 °C.

## 4. Conclusions

Herein, we presented a successful preparation of a cellulose scaffold/ANF hybrid bulk composite with outstanding mechanical performance and thermal stability through delignification, ANF impregnation and densification. Air drying process of delignified wood brings a dense structure, leading to improved mechanical properties (2.5 times for tensile strength, 2.5 times for toughness and 2.3 times for Young’s modulus) as compared to natural wood. The densification of cellulose scaffold impregnated with ANF endowed cellulose scaffold/ANF hybrid bulk composite with a more compact cement-like microstructure, inducing a superior mechanical properties and thermal stability. The 341.7 MPa tensile strength, 4.4 MJ/m^3^ toughness and 24.7 GPa Young’s modulus of cellulose microfibers/ANF hybrid bulk material approximately 5, 10 and 2.4 times higher, respectively, as compared to those of natural wood. The above results indicated that ANF played a very important role in assisting the assembly of cellulose scaffold into a good thermal stability and superior mechanical material, by forming hydrogen bonding with cellulose microfibers under compression. The product could be used in the design of high-performance biobased hybrid material for application in wooden architecture, automotive equipment and spacecraft.

## Data Availability

The data presented in this study are available upon request from the corresponding author.

## Supplementary Materials

The following are available online at https://www.mdpi.com/article/10.3390/ma14185444/s1. Figure S1: The preparation of aramid nanofiber (ANF) and molecular structure of ANF, Figure S2: Delignification process of natural wood by NaClO_2_ and NaOH treatment, Figure S3: SEM images of NW, Figure S4: SEM transverse images of CS_AD_, Figure S5: SEM longitudinal images of CS_AD_, Figure S6: SEM transverse images of CCS_AD-ANF_ (a, b) and SEM longitudinal images of CCS_AD-ANF_ (c, d), Figure S7: Magnified FTIR spectra for CS and CCS_AD-ANF_.
