# Supplementary material for: Hydrogen-Bonding-Aided Fabrication of Wood Derived Cellulose Scaffold/Aramid Nanofiber into High-Performance Bulk Material
Source: Materials (Basel). 2021 Sep 20;14(18):5444. doi: 10.3390/ma14185444 (PMC8469447; doi:10.3390/ma14185444)
Supplement: Supplementary file 1 [file materials-14-05444-s001.zip › materials-1378584-supplementary.pdf]

materials

Supplementary Materials

Hydrogen-Bonding-Aided Fabrication of Wood Derived Cellulose Scaffold/Aramid Nanofiber into High-Performance Bulk Material

Xiaoshuai Han, Weijie Wu, Jingwen Wang, Zhiwei Tian and Shaohua Jiang *

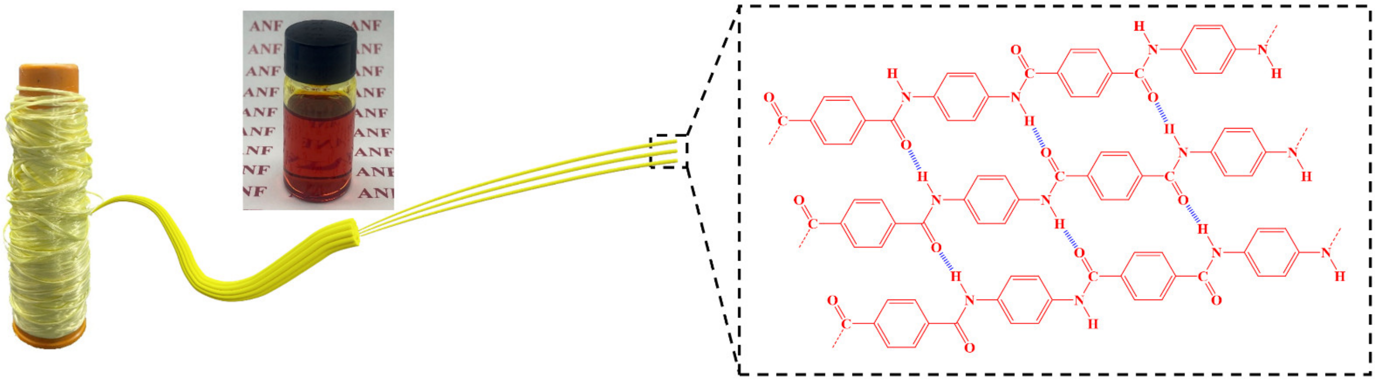

Figure S1. The preparation of aramid nanofiber (ANF) and molecular structure of ANF.

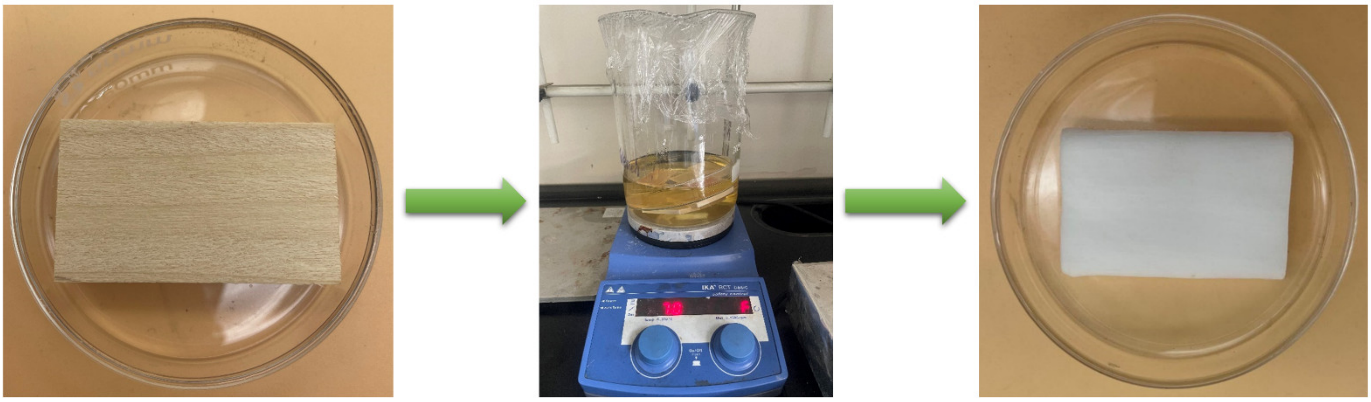

Figure S2. Delignification process of natural wood by $NaClO_2$ and NaOH treatment.

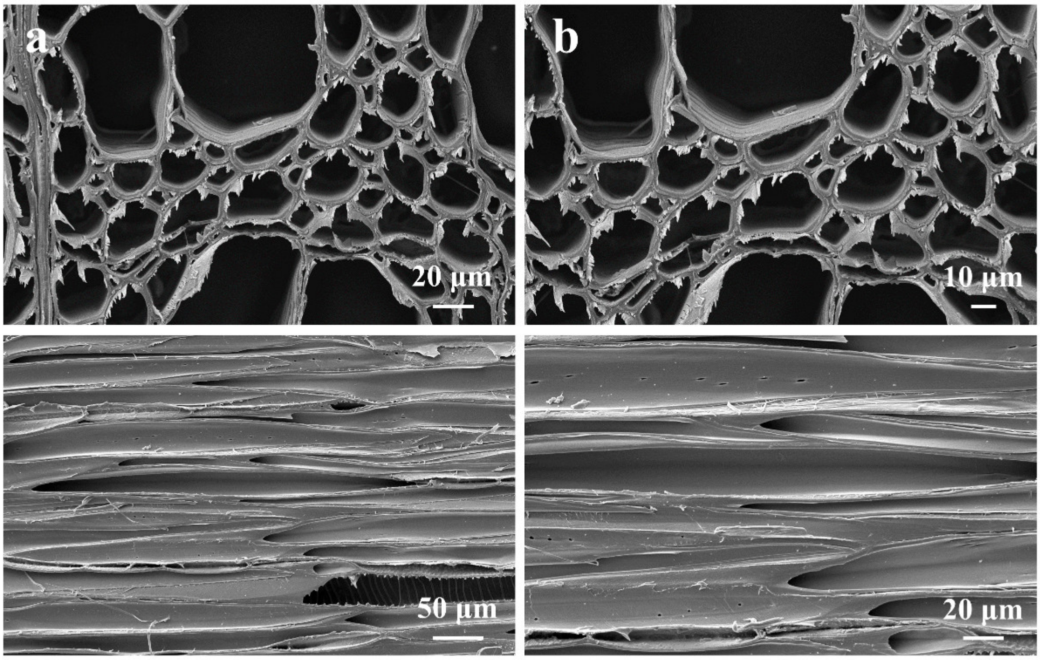

Figure S3. SEM images of NW.

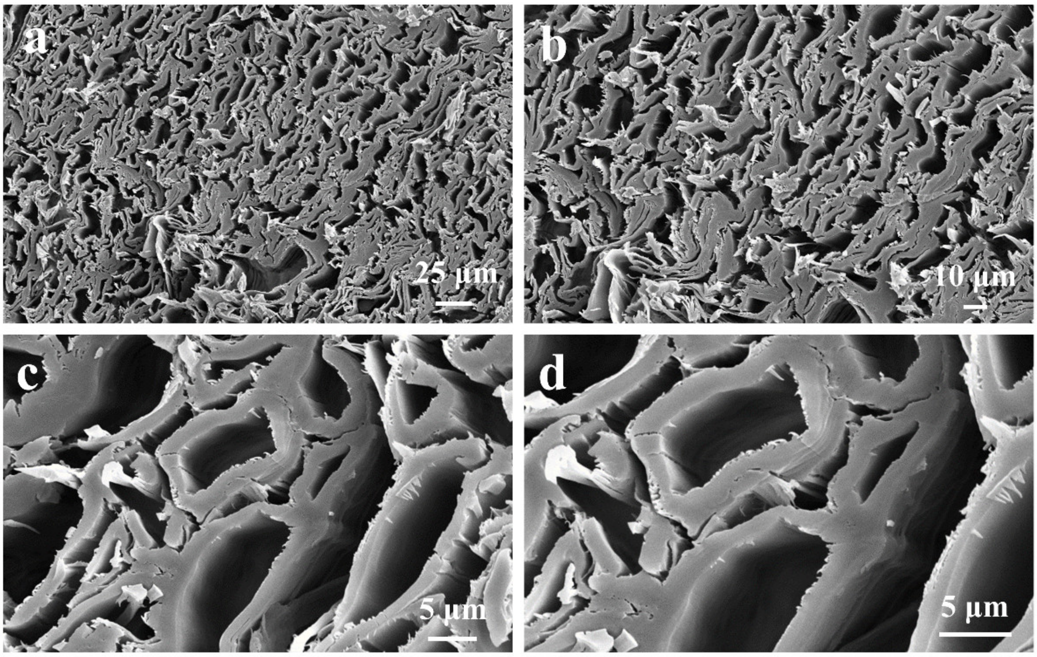

Figure S4. SEM transverse images of CS_{AD}.

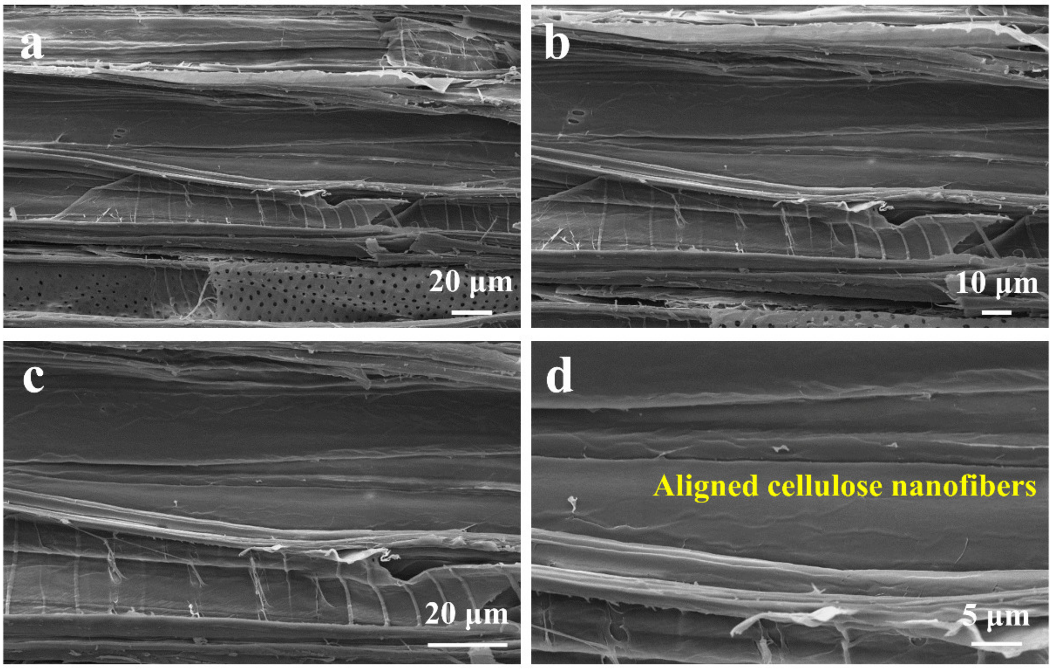

Figure S5. SEM longitudinal images of CS_{AD}.

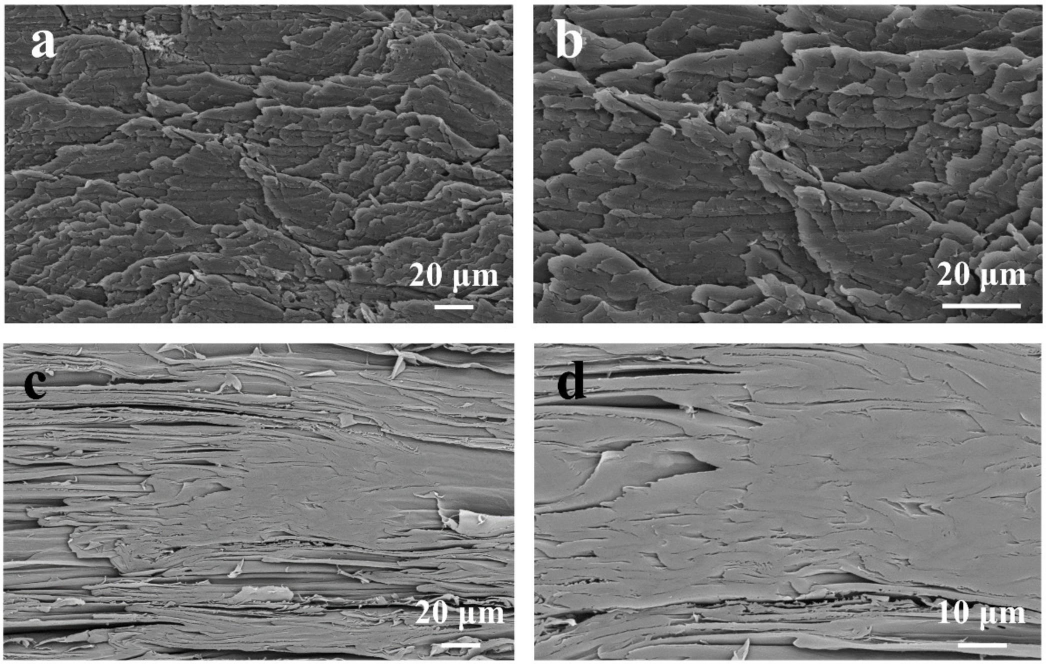

Figure S6. SEM transverse images of CCS_{AD-ANF} (a, b) and SEM longitudinal images of CCS_{AD-ANF} (c, d).

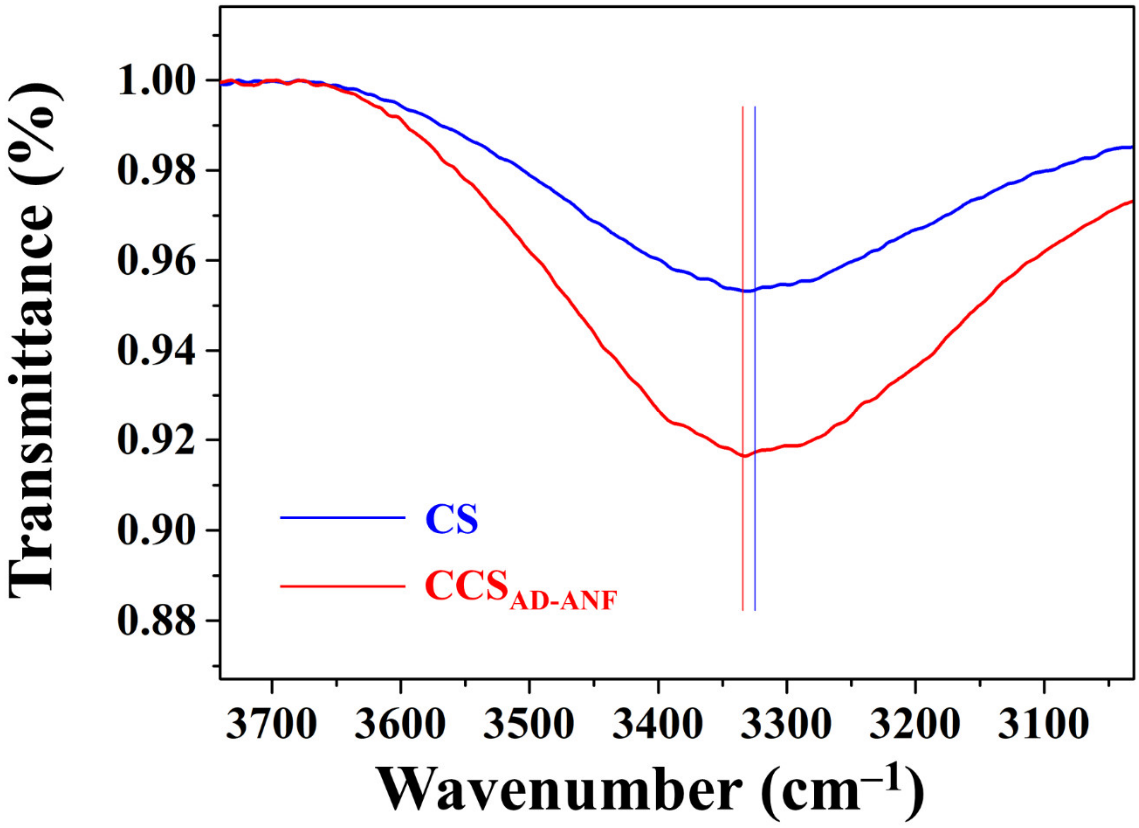

Figure S7. Magnified FTIR spectra for CS and CCSAD-ANF
